# 5-HT6 receptor agonism facilitates emotional learning

**DOI:** 10.3389/fphar.2015.00200

**Published:** 2015-09-16

**Authors:** Marcela Pereira, Bruno J. Martynhak, Roberto Andreatini, Per Svenningsson

**Affiliations:** ^1^Section of Translational Neuropharmacology, Department of Clinical Neuroscience, Center of Molecular Medicine, Karolinska InstituteStockholm, Sweden; ^2^Department of Pharmacology, Federal University of ParanáCuritiba, Brazil

**Keywords:** 5-HT6, antidepressant, memory, passive avoidance, forced swim test, c-fos, MAPK

## Abstract

Serotonin (5-HT) and its receptors play crucial roles in various aspects of mood and cognitive functions. However, the role of specific 5-HT receptors in these processes remains to be better understood. Here, we examined the effects of the selective and potent 5-HT6 agonist (WAY208466) on mood, anxiety and emotional learning in mice. Male C57Bl/6J mice were therefore tested in the forced swim test (FST), elevated plus-maze (EPM), and passive avoidance tests (PA), respectively. In a dose-response experiment, mice were treated intraperitoneally with WAY208466 at 3, 9, or 27 mg/kg and examined in an open field arena open field test (OFT) followed by the FST. 9 mg/kg of WAY208466 reduced immobility in the FST, without impairing the locomotion. Thus, the dose of 9 mg/kg was subsequently used for tests of anxiety and emotional learning. There was no significant effect of WAY208466 in the EPM. In the PA, mice were trained 30 min before the treatment with saline or WAY208466. Two separate sets of animals were used for short term memory (tested 1 h post-training) or long term memory (tested 24 h post-training). WAY208466 improved both short and long term memories, evaluated by the latency to enter the dark compartment, in the PA. The WAY208466-treated animals also showed more grooming and rearing in the light compartment. To better understand the molecular mechanisms and brain regions involved in the facilitation of emotional learning by WAY208466, we studied its effects on signal transduction and immediate early gene expression. WAY208466 increased the levels of phospho-Ser^845^-GluA1 and phospho-Ser^217/221^-MEK in the caudate-putamen. Levels of phospho-Thr^202/204^-Erk1/2 and the ratio mature BDNF/proBDNF were increased in the hippocampus. Moreover, WAY208466 increased c-fos in the hippocampus and Arc expression in both hippocampus and prefrontal cortex (PFC). The results indicate antidepressant efficacy and facilitation of emotional learning by 5-HT6 receptor agonism via mechanisms that promote neuronal plasticity in caudate putamen, hippocampus, and PFC.

## Introduction

Brain serotonin (5-HT) is implicated in a wide variety of physiological functions related to mood, cognition and movements. The mechanisms whereby 5-HT6 and its receptors exert its versatile functions are complex and often contradictory. For example, 5-HT6 agonists (Svenningsson et al., 2007; Carr et al., 2011; Kendall et al., 2011) and antagonists (Hirst et al., 2006; Wesolowska and Nikiforuk, 2007, 2008; Hirano et al., 2009) have procognitive and/or antidepressant-like effects in animal models. Nonetheless, since many serotonergic compounds have entered, or are about to enter, the clinics, it is critically important to better delineate action of serotonergic compounds. This is particularly evident for 5-HT6 ligands as the combination of donepezil with a 5-HT6 antagonist, idalopirdine, improved the cognitive function of patients with Alzheimer’s Disease (Wilkinson et al., 2014).

The exact role of 5-HT6 receptor activation for memory acquisition and consolidation is not yet completely understood. Virally mediated gene transfer to overexpress 5-HT6 receptors in the striatum had no effect on performance in the Morris water maze (hippocampus-dependent), but impaired the acquisition of a reward-based instrumental learning task (striatum-dependent), an effect rescued by treatment with the 5-HT6 antagonist, SB-258585 (Mitchell et al., 2007). However, treatment with the 5-HT6 agonist, WAY181187, facilitated extra-dimensional attentional set shifting [prefrontal cortex (PFC)-dependent] and increased c-fos expression in the PFC (Burnham et al., 2010). Administration of the 5-HT6 agonists, E-6801, or EMD-386088, reversed the cognitive deficits induced by scopolamine or MK-801 pretreatment in the conditioned emotion response, a cued and contextual fear memory (hippocampal, amygdala, and cortical-dependent; Woods et al., 2012). Paradoxically, both administration E-6801 and EMD-386088 as well as the 5-HT6 antagonists, SB-271046 and Ro 04–6790, improved the recognition memory (hippocampal-dependent; Kendall et al., 2011).

The PA test evaluates emotional memory (Burwell et al., 2004; Mitchell and Neumaier, 2005; Eriksson et al., 2008). PA is considered a complex memory test since it is comprised by both a Pavlovian component and also requires an instrumental response. In this test, animals are required to suppress the natural preference of a dark compartment to avoid a foot shock (e.g., Baamonde et al., 1992; Ogren et al., 2008). PA is hippocampal dependent and several studies have shown the importance of serotonin in this test (Misane and Ogren, 2000; Eriksson et al., 2008, 2013). The role of 5-HT6 agonists in PA is unknown, but other hippocampal dependent memories are modulated by 5-HT6 agonists (Kendall et al., 2011; Woods et al., 2012) and 5-HT6 antagonists (Lieben et al., 2005; Meneses et al., 2007; Kendall et al., 2011; Woods et al., 2012).

We have previously shown that the 5-HT6 agonist 2-ethyl-5-methoxy-N, *N*-dimethyltryptamine (EMDT), similarly to fluoxetine, induces antidepressant effect in the mouse tail suspension test and increases the phospho-Ser^845^-GluA1 subunit of the AMPA receptor in the PFC and striatum (Svenningsson et al., 2007). Interestingly, the 5-HT6 antagonist, SB271046, blocked not only the effects of EMDT and but also counteracted effects of fluoxetine (Svenningsson et al., 2007).

The objective of this study was to further evaluate emotional processing along with antidepressant and anxiolytic actions by the highly selective and potent 5-HT6 agonist, WAY208466 (Schechter et al., 2008). Moreover, to understand molecular mechanisms of action and brain regions engaged by WAY208466, we also evaluated its effects on signal transduction and immediate early genes (IEGs) involved in neuronal plasticity. The roles of many IEGs are indeed related to neuroplasticity (Pei et al., 2004). Here we studied representative genes from two classes, a transcription factor (i.e., c-fos) and an effector (i.e., Arc – activity-regulated cytoskeletal associated gene; Clayton, 2000).

## Materials and Methods

### Animals

Adult male C57Bl/6J mice were obtained from Janvier labs (Scand-las Turku, Finland) and housed under controlled temperature and humidity with food and water *ad libitum* and in a 12 h light/dark controlled cycle. All experiments were carried out in agreement with the European Council Directive (86/609/EEC) and were approved by the local Animal Ethics Committee (N40/13; Stockholm Norra Djurförsöksetiska Nämnd). All efforts were made to reduce the number of animals used and to minimize their suffering.

### Treatment

For all behavioral testing, animals were brought to the experimental room 30 min for habituation. Animals then received a single intraperitoneal injection of WAY208466 (3-[(-3-Fluorophenyl)sulfonyl]-*N, N*-dimethyl-1*H*-pyrrolo[2,3-*b*]pyridine-1-ethanamine dihydrochloride; Tocris Bioscience, Bristol, UK) or vehicle (saline) 30 min prior to behavioral tests. In an initial dose-response experiment, we examined three different doses (3, 9, and 27 mg/kg) of WAY208466 in the OFT and in the FST. Since the dose of 27 mg/kg impaired locomotor activity in the OFT and the dose of 3 mg/kg did not reduce the immobility in the FST, we used 9 mg/kg for subsequent tests (i.e., PA and EPM). In addition, and for comparison, naïve groups were treated with vehicle or 9 mg/kg of WAY208466 and euthanized.

### Behavioral Tests

#### Forced Swim Test

The Porsolt forced swim test (FST) procedure was performed as described earlier (Cervo et al., 2005). Animals were individually placed in a vertical Plexiglas cylinder (height: 30 cm, diameter: 20 cm) filled with 15 cm depth water at 23–25°C. The water was changed between every animal. The animals were removed from the water after 6 min, and dried before they returned to their home cages. Behavior was analyzed in the last 4 min of the test (Guzzetti et al., 2008). The experiment was recorded and analyzed automatically using NOLDUS Ethovision XT9 software (Wageningen, The Netherlands).

#### Open Field Test

Mice were tested in the OFT for 5 min. The open field arena (46 cm × 46 cm) was illuminated by a reflected light of approximately 35 lux. Performance in the OFT was tracked and analyzed using an automated video tracking system (NOLDUS Ethovision XT9, Wageningen, The Netherlands).

#### Passive Avoidance Test

The step-through passive avoidance (PA) was performed as described earlier (Eriksson et al., 2013). Briefly, the PA apparatus (25 cm × 50 cm × 25 cm) consisted of two equally sized compartments connected by a sliding door (7 cm × 7cm) (Ugo Basile, Comerio-Varese, Italy). The light intensities in the dark and the bright compartments were 2 and 250 lx, respectively. During PA training, each mouse was placed in the bright compartment and allowed to explore it for 60 s. The sliding door was then opened and the animal had a maximum of 300 s to step through to the dark compartment. Once the mouse had entered the dark compartment, the sliding door was automatically closed and, after 3 s, a weak electrical stimulus (0.3 mA, 2 s scrambled current) was delivered through the grid floor.

After 1 h short term memory (STM) or 24 h long term memory (LTM), the animal was again gently placed in the light compartment, and the latency to enter the dark compartment with all four paws was measured (retention latency) with a 9 min cutoff time for testing. No electrical stimulus was given during the second exposure. The parameters evaluated were retention latency, grooming, and rearing. All parameters were observed and registered manually during the experiment (latency to step through, grooming and rearing). The animals were euthanized 1 h after the test and their brains were later used for the *in situ* hybridization (described in Section “Immunoblotting”).

#### Elevated Plus-Maze

The elevated plus-maze (EPM) was conducted as previously described (Kindlundh-Högberg et al., 2009). Mice were placed in the center facing an open arm and allowed to explore the apparatus for 5 min. Entries into the open (90 lux) and closed (20 lux) arms and time spent in each arm were measured by automated video tracking system (NOLDUS Ethovision XT9, Wageningen, The Netherlands). The animals were euthanized 1 h after the test and their brains were later used for the immunoblotting (described in Section “*In Situ* Hybridization”).

### Immunoblotting and Histological Measurements

#### Tissue Collection

Mice were sacrificed by decapitation; their brains were quickly dissected and dipped in isopentane, cooled in dry ice, for approximately 5 s. Samples were stored in -80°C freezer for further processing.

#### *In Situ* Hybridization

Fresh frozen coronal cryostat sections (14 μm) were prepared and hybridized with ^35^S-radiolabeled antisense riboprobes against Arc and c-fos. The sections were exposed to Kodak MR film in room temperature for 7–21 days prior to development, according to a previously published protocol (Svenningsson et al., 1998, 2006). The areas selected for analysis were the PFC, the striatum/CPu, the nucleus accumbens (NAcc), the amygdala (basolateral nuclei of amygdala –LA/BLA), and the hippocampus (Hi – four different subareas: *Cornu Ammonis* – subareas CA1, CA2, CA3, and dentate gyrus – DG). Densitometric measurements were obtained from autoradiograms using the NIH ImageJ 1.40 software (National institute of Mental Health, Bethesda, MD, USA). All optical density values were normalized. For each target analyzed, the average of the control group (naïve group treated with saline) was normalized to 100% and results from each treatment group are presented as percentage of the control.

#### Immunoblotting

Tissues from PFC, hippocampus, and caudate-putamen (CPu) were sonicated and boiled in 1% sodium dodecyl sulfate (SDS) containing a protease and phosphatase inhibitor Cocktail (HaltTM, Pierce, Rockford, IL, USA). Protein concentration was determined in each sample using a bicinchoninic acid protein assay (BCA-kit, Pierce, Rockford, IL, USA). Equal amounts of protein (5–20 μg) were separated by SDS–polyacrylamide gel electrophoresis using 8% lower running gels. Proteins were transferred to Immobilon-P (Polyvinylidene Difluoride) membranes (Millipore, Bedford, MA, USA). Membranes were blocked by incubation in 5% (w/v) dry milk or bovine serum albumin (BSA) in TBS-Tween20 for 1 h at room temperature. Following overnight incubation with primary antibodies (**Table 1**), the membranes were washed three times with TBS-Tween 20 and incubated for 1 h with secondary horseradish peroxidase (HRP)-linked Anti-Rabbit IgG (H + L) (Dako, Glostrup, Denmark). Immunoreactive bands were detected by enhanced chemiluminescence (Bio-Rad, Bio-Rad, Hercules, CA, USA) and quantified by densitometry with ImageJ 1.40 software. All data are presented as values normalized to the levels of β-actin or calnexin. The level of the phosphorylated form of a protein was normalized to the total level of the same protein. For each target analyzed, the average of the saline group was normalized to 100% and results from each treatment group are presented as percentage of the saline group.

**Table 1:** List of the antibodies and dilutions used for the immunoblotting.

Antibody	Company	Catalog number	Dilution
Actin	Sigma	A5060	1:10000
Calnexin	Sigma	C4731	1:2000
Glu R1	Milipore	06–306	1:1000
P-Ser^845^ Glu R1	Milipore	04–823	1:1000
MEK	Cell signaling	9122	1:1000
P-Ser^217/221^ MEK	Cell signaling	9121	1:1000
Erk1/2	Cell signaling	9107S	1:2000
P-Thr^202/204^ Erk1/2	Cell signaling	9101S	1:1000
proBDNF	Alomone	ANT-006	1:200
mBDNF	Sigma	AV-41970	1 μg/ml

### Statistical Analysis

Data were initially evaluated for outliers with the Grubb’s test. Immunoblotting and behavior in the PA test and were analyzed with Student’s *t*-test. Dose-response effects of WAY208466 on the OFT and FST were analyzed by one-way analysis of variance (ANOVA) with treatment as a factor. Arc and c-fos expression were analyzed with two-way ANOVA with training × treatment as factors. ANOVAs were followed by Fisher’s least significance difference (LSD) *post hoc* test. All data are presented as mean ± SEM and significance was defined as *p* < 0.05.

## Results

### Behavioral Tests

#### Forced Swim Test

**FIGURE 1:**
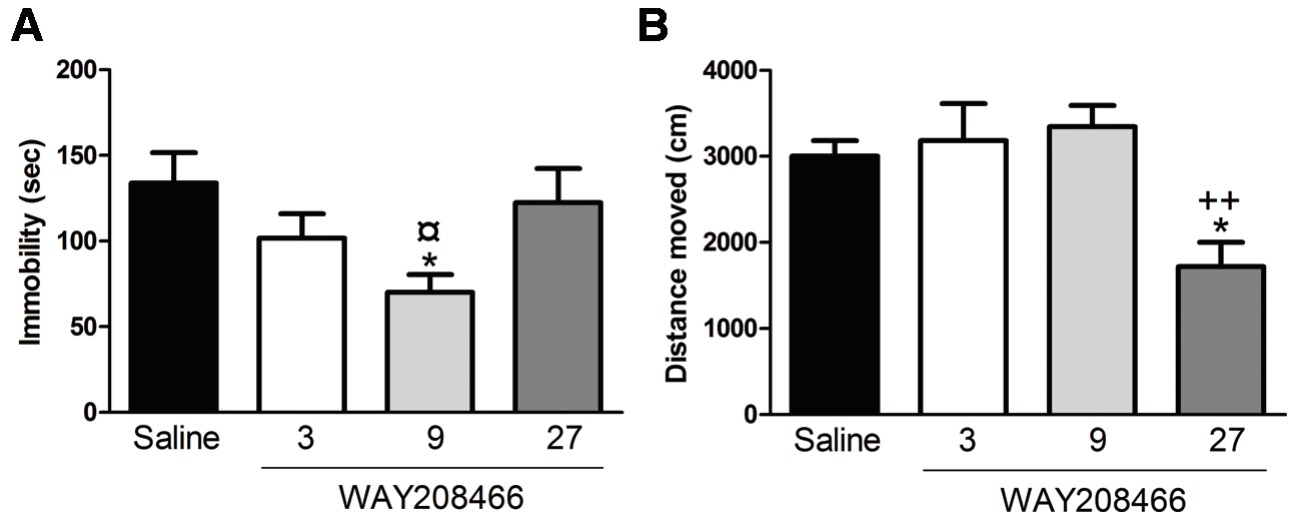
**Effects of WAY208466 treatment in the forced swim test (FST) and open field test (OFT). (A)** Immobility duration in the FST showing antidepressant-like effect of WAY208466 after 9 mg/kg (ip). **(B)** Total distance moved during 5 min in the OFT. WAY208466 at 27 mg/kg (ip) caused motor impairment compared to the saline group. ^∗^*p* < 0.05 compared to saline group, ^¤^*p* < 0.05 compared to 27 mg/kg. ^++^*p* < 0.01 compared to 9 mg/kg WAY208466 group. Mean ± SEM; *n* = 8 mice per group.

Analysis with one-way ANOVA showed a statistical difference between treatments (*F*_3,28_= 3.045; *p* < 0.05). Fisher *post hoc* analysis showed that 9 mg/kg of WAY208466 decreased the immobility (**Figure 1A**).

#### Open Field Test

One-way ANOVA showed a statistical difference between treatments (*F*_3,28_= 6.70, *p* < 0.01). WAY208466, at the highest dose (27 mg/kg), decreased locomotion (**Figure 1B**).

#### Passive Avoidance Test

**FIGURE 2:**
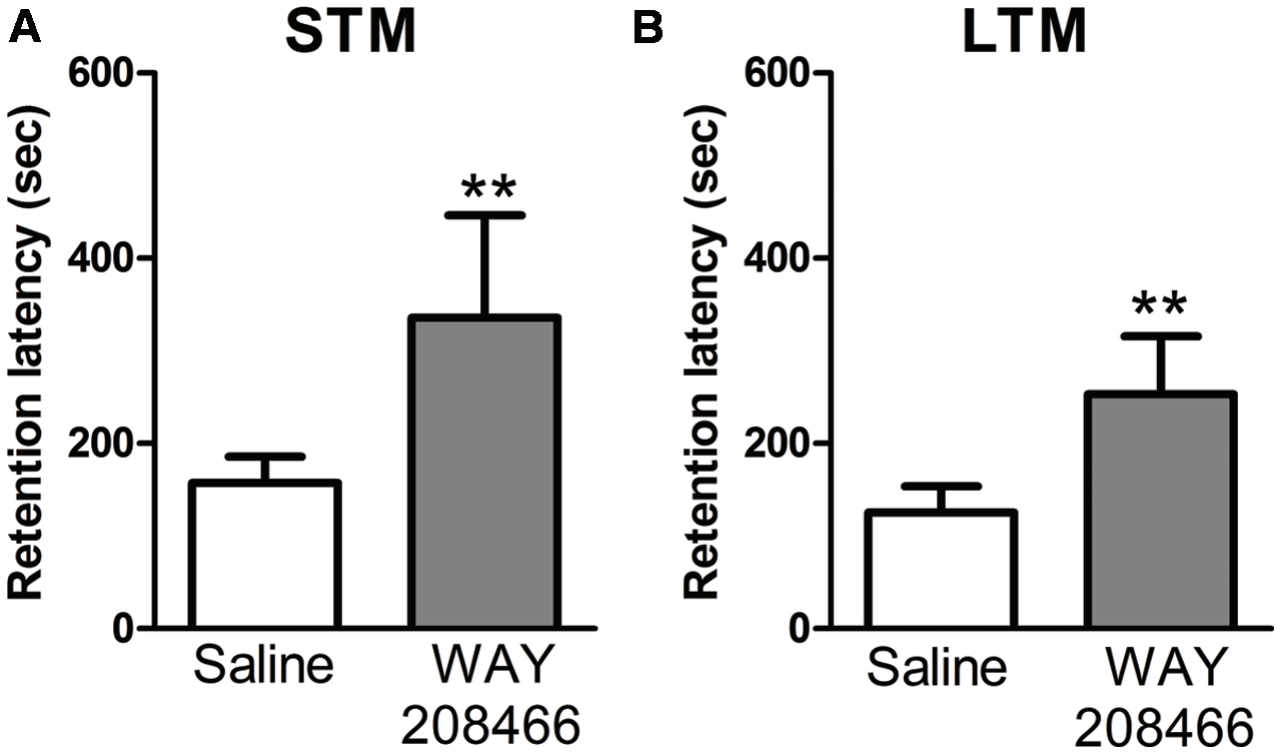
**Retention latency in the PA test.** Animals were treated with saline or WAY208466 (9 mg/kg), 30 min prior test (ip). **(A)** Short term (1 h) memory (STM) comparison between treatments. **(B)** Long term (24 h) memory (LTM) comparison between treatments. ^∗∗^*p* < 0.01 compared to saline group. Mean ± SEM, *n* = 8 mice per group.

No significant differences were observed between the saline group and WAY208466 (9 mg/kg) during the training session for either short term or long term memories in the latency to step through to the dark compartment (*t* = -1.44, *p* < 0.17; *t* = 0.29, *p* < 0.2, respectively). However, during the test section, student *t*-test showed a significant difference for both short (*t* = -2.21, *p* < 0.05) and long (*t* = -2.64, *p* < 0.01) term memories (**Figure 2**).

**FIGURE 3:**
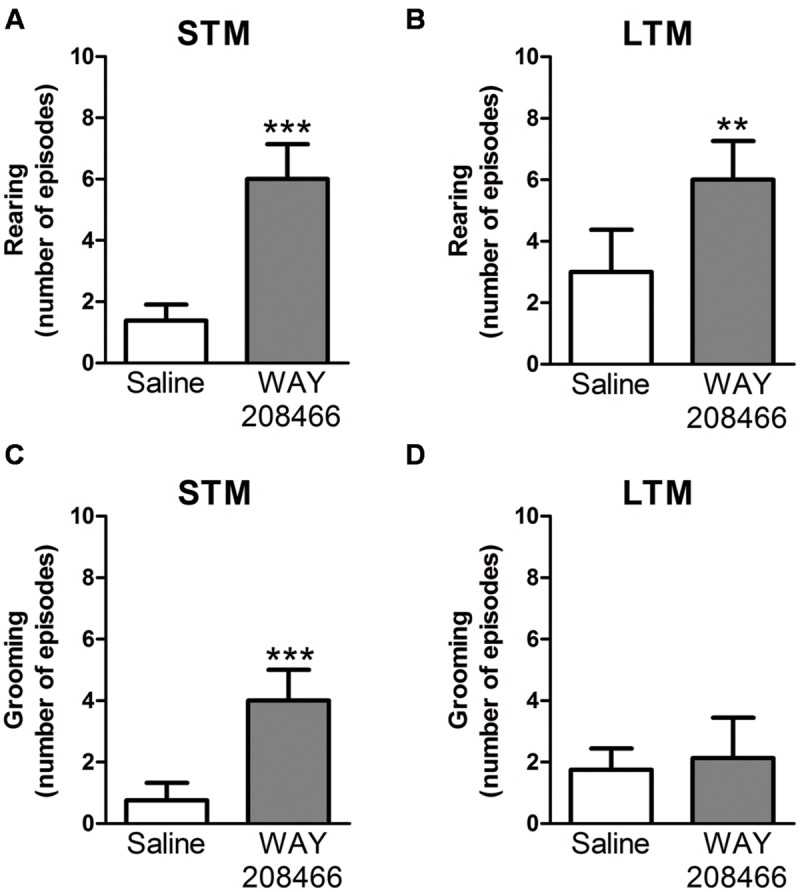
**Rearing **(A,B)** and grooming **(C,D)** during PA test.** Differences in the number of rearings between treatments [saline or WAY208466 (9 mg/kg)] for STM **(A)** or LTM **(B)**. Differences in the frequency of groomings between treatments [saline or WAY208466 (9 mg/kg)] for STM **(C)** or LTM **(D)**. ^∗∗^*p* < 0.01; ^∗∗∗^*p* < 0.001 compared to saline group. STM, short term memory; LTM, long term memory. Mean ± SEM; *n* = 8 mice per group.

During the PA test, grooming and rearing were also examined. No significant differences were observed during training sessions for either grooming (*t* = -1.55, *p* < 0.14; *t* = -0.17, *p* < 0.87, respectively) or rearing (*t* = 0.40, *p* < 0.69; *t* = -0.17, *p* < 0.87, respectively). However, rearing was significant increased by WAY208466 (9 mg/kg) both in the test sessions for short and long term memories (*t* = -5.22, *p* < 0.001; *t* = -3.55, *p* < 0.01, respectively). Grooming was increased by WAY208466 in the STM paradigm (*t* = -3.97, *p* < 0.01), but not in LTM (*t* = -0.36, *p* < 0.72) (**Figure 3**).

#### Elevated Plus-Maze

No significant differences were observed between the saline group and WAY208466 (9 mg/kg) in the EPM test. Student’s *t*-test showed no differences in neither number of entries in the open arm of the maze (*t* = 1.59, *p* < 0.13), nor in time spent in the open arm (*t* = 1.66, *p* < 0.11) (**Table 2**).

**Table 2:** Effects of saline and WAY208466 (9 mg/kg) treatment in the EPM test.

Treatment	Entries in the open arms	Time (sec) spent in the open arms
Saline	18.6 ± 0.92	117.5 ± 12.8
WAY208466	17.0 ± 1.10	99.11 ± 8.97

*Data represented as mean ± SEM, *n* = 8 mice per group.*

### Immunoblotting and Histological Measurements

#### *In Situ* Hybridization

No significant changes in c-fos or Arc expression were observed in any of the analyzed areas (PFC, CPu, NAccs, amygdala, and hippocampus) in animals studied in the STM paradigm of PA.

**FIGURE 4:**
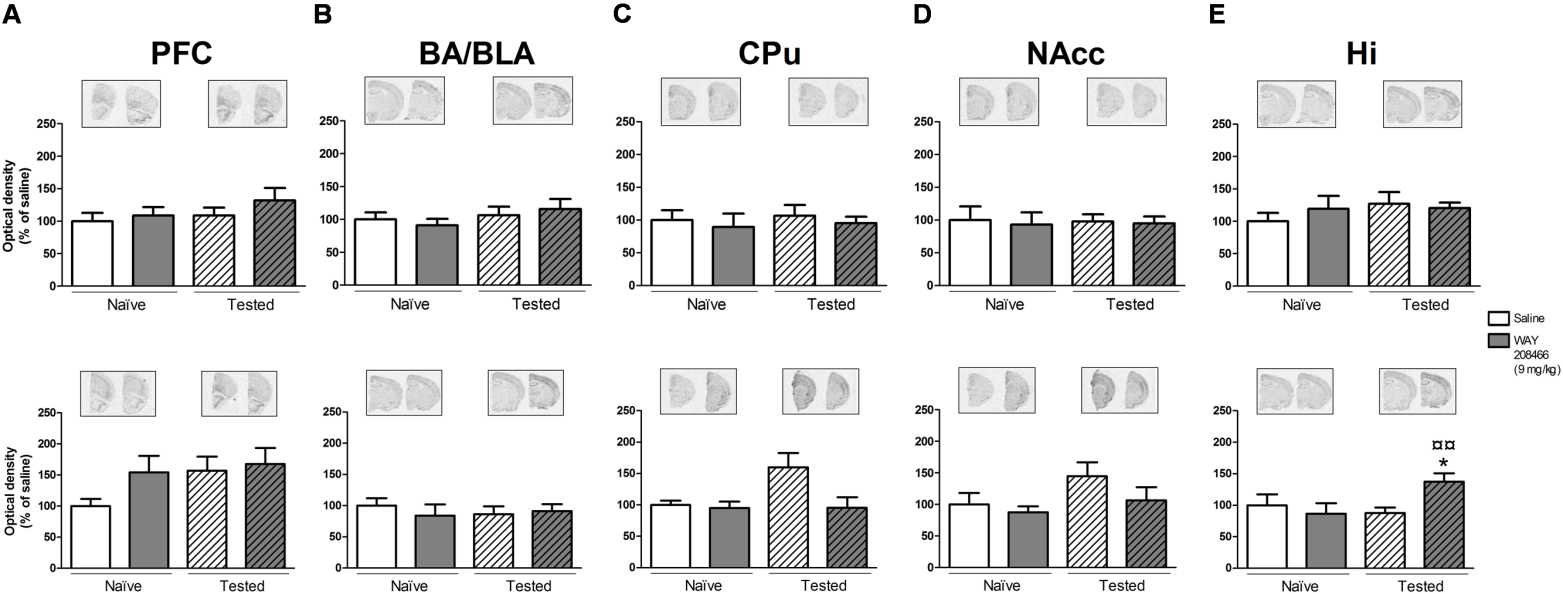
**Effects of saline and WAY208466 (9 mg/kg, ip) on the levels of c-fos mRNA after PA test for STM (upper) or LTM (lower) for **(A)** PFC, pre frontal cortex; **(B)** BA/BLA, lateral and basolateral amygdala; **(C)** CPu, caudate putamen; **(D)** NAcc, nucleus accumbens; **(E)** Hi, hippocampus.** ^∗^*p* < 0.05 compared to saline/naïve group. ^¤^*p* < 0.05; ^¤¤^*p* < 0.01 compared to saline group. Mean ± SEM; *n* = 6–8 mice per group.

**FIGURE 5:**
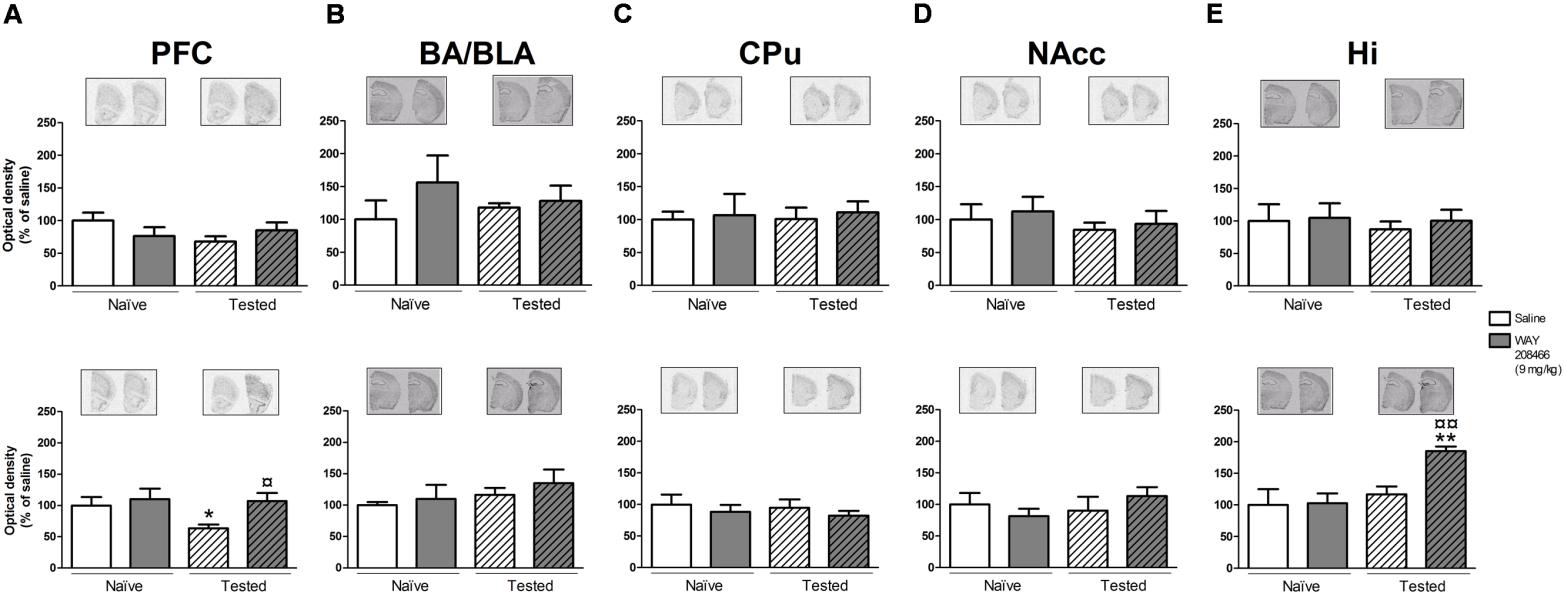
**Effects of saline and WAY208466 (9 mg/kg, ip) on the levels of Arc (activity-regulated cytoskeleton-associated protein) mRNA after PA test for STM (upper) or LTM (lower) for **(A)** PFC, pre frontal cortex; **(B)** BA/BLA, lateral and basolateral amygdala; **(C)** CPu, caudate putamen; **(D)** NAcc, nucleus accumbens; **(E)** Hi, hippocampus.** ^∗^*p* < 0.05; ^∗∗^*p* < 0.01 compared to saline naïve group. ^¤¤^*p* < 0.01 compared to saline group. Mean ± SEM; *n* = 6–8 mice per group.

However, in the LTM PA paradigm, two-way ANOVAs followed by Fisher’s *post hoc* test showed increases of both hippocampal c-fos and Arc mRNAs in tested animals treated with WAY208466 in comparison with either saline/trained (*p* < 0.05 and *p* < 0.01, respectively) or treated/naïve (*p* < 0.05, *p* < 0.01, respectively) groups. (c-fos: treatment: *F*_1,21_ = 1.88, *p* < 0.05, training *F*_1,21_ = 2.08, *p* > 0.05, treatment × training interaction: *F*_1,21_ = 5.26, *p* < 0.05; Arc: treatment: *F*_1,21_ = 5.67, *p* < 0.05, training: *F*_1,21_ = 11.10, *p* < 0.01, treatment × training interaction: *F*_1,21_= 4.82, *p* < 0.05) (**Figures 4E and 5E**).

A two-way ANOVA also detected a treatment effect of WAY208466 in the Arc expression in the PFC in the LTM paradigm (treatment: *F*_1,21_ = 4.75, *p* < 0.05, training: *F*_1,21_ = 2.56, *p* > 0.05, treatment × training interaction: *F*_1,21_ = 0.12, *p* > 0.05). Specifically, WAY208466 treatment prevented the reduction in Arc expression observed in trained animals in comparison with the naïve groups (*p* < 0.05) (**Figure 5A**).

No significant changes were observed for the other analyzed areas in the long term memory paradigm (**Figures 4A–D** and **5B–D**).

#### Western Blot

**FIGURE 6:**
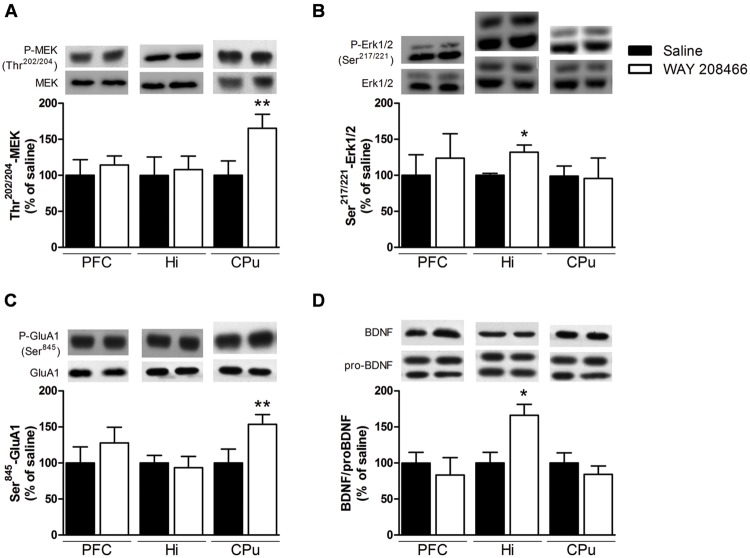
**Effects of saline or WAY208466 (9 mg/kg, ip) on the phosphorylation states of Ser^217/221^-MEK **(A)**, Thr^202/204^-Erk1/2 **(B)**, Ser^845^-GluA1 **(C)** and the ratio matureBDNF/proBDNF **(D)** in the prefrontal cortex (PFC), hippocampus, and caudate-putamen (CPu).** ^∗^*p* < 0.05; ^∗∗^*p* < 0.01 compared to saline group. Mean ± SEM; *n* = 6–8 mice per group.

Acute treatment with WAY208466 (9 mg/kg) increased the levels of phospho-Ser^845^-GluA1 in the CPu (*t* = 2.21, *p* < 0.05), but not in the PFC or hippocampus (*t* = 0.88, *p* > 0.2; *t* = 0.35, *p* > 0.2, respectively) (**Figure 6C**). Similarly, phospho-Ser^217/221^MEK was also increased in the CPu (*t* = 2.31, *p* < 0.05), but not in the PFC or hippocampus (*t* = 1.03, *p* > 0.2; *t* = 0.68, *p* > 0.2, respectively) (**Figure 6A**).

Levels of phospho-Thr^202/204^Erk1/2 were increased in the hippocampus (*t* = 2.71, *p* < 0.05), but not in the PFC or CPu (*t* = 0.33, *p* > 0.2; *t* = 0.10, *p* > 0.2, respectively) (**Figure 6B**). Likewise, the ratio of mature BDNF/proBDNF was increased in the hippocampus (*t* = 2.71, *p* < 0.05), but not in the PFC or CPu (*t* = 0.95, *p* > 0.2, respectively) (**Figure 6D**).

## Discussion

Our results show that the 5-HT6 agonist WAY208466 facilitates cognitive processing in the PA test and has an antidepressant-like effect in the FST. In agreement with a previous study performed in rats (Carr et al., 2011), we observed a U-shape dose-response curve in the antidepressant effect of WAY208466. The study of Carr et al. (2011) used rats as they have higher expression of 5-HT6 receptors when compared to mice (Hirst et al., 2003; Zhang et al., 2011). Nonetheless, our data demonstrates antidepressant properties of WAY208466 also in mice.

In addition of improving the performance in the PA test, WAY208466 also increased grooming and rearing during this test. Grooming/rearing in a new environment interacts with anxiety in a complex manner (Prut and Belzung, 2003). Furthermore, since Carr et al. (2011) found an anxiolytic-like effect of WAY208466, we performed experiments in EPM. Somewhat surprisingly, we did not find a significant effect of WAY208466 in the EPM. It is important to note that the absence of effect in our experiment might be related to the low sensitive of the EPM test to serotoninergic drugs.

The OFT was performed to evaluate for possible locomotor effects induced by WAY208466 that could bias the subsequent behavioral tests. We found that 27 mg/kg of WAY208466 reduced locomotion in the OFT. This result is inconsistent with a previous report (Carr et al., 2011), in which 30 mg/kg of WAY208466 caused no hypolocomotion in rats. The discrepancy between these results may, at least partly, be explained by different experimental designs. Carr et al. (2011) treated rats for 1 h before examining their locomotion for 30 min, whereas we treated mice for 30 min before examining their locomotion for 5 min. A possible explanation for the different results is that our experiments are strongly influenced by a novelty response together with regular locomotor activity, whereas the results from Carr et al. (2011) were less influenced by novelty. Based on this result in the OFT we decided to not perform additional experiment with 27 mg/kg of WAY208466. 3 and 9 mg/kg of WAY208466 caused no hypolocomotion, but only 9 mg/kg decreased immobility in the FST. Based on these results, the subsequent EPM and PA experiments were only performed using 9 mg/kg of WAY208466.

To better understand the molecular mechanisms and brain regions involved in the facilitation of emotional learning by WAY208466, we correlated its behavioral effects with alterations on signal transduction and IEG expression. Studies have reported the importance of mitogen-activated protein kinase (MAPK) signaling pathway in the process of memory consolidation (Thomas and Huganir, 2004). We observed that WAY208466 increased the phosphorylation of MEK in the CPu and Erk1/2 in the hippocampus. Interestingly, treatment with the clinically used procognitive agent, memantine, has also been shown to both improve the PA performance and to increase hippocampal Erk1/2 phosphorylation (Liu et al., 2014).

Corroborating with our previous results using EMDT (Svenningsson et al., 2007), treatment with WAY208466 increased phosphorylation of the Ser^845^-GluA1 receptors in the CPu. Phosphorylation of Ser^845^-GluA1 in the ventral striatum has been reported to be important for spatial memory consolidation (Ferretti et al., 2014). The role of the CPu for learning the PA task is not as evident as that of hippocampus, although cholinergic blockade in the CPu impairs the memory formation in the PA test (Prado-Alcalá et al., 1985). Unlike our previous report with EMDT, (Svenningsson et al., 2007) WAY208466 treatment did not increase Ser^845^-GluA1 phosphorylation in the PFC.

BDNF is critically important in multiple plastic changes regulating mood and cognition and was therefore also studied in the immunoblotting experiments. Treatment with WAY208466 did not change mature BDNF or proBDNF levels in hippocampus. However, the ratio of mature BDNF/proBDNF was increased, favoring neuronal plasticity.

To determine brain regions affected by the PA paradigm and 5-HT6 agonism, we evaluated the expression of the IEGs Arc and c-fos by *in situ* hybridization. Since the initial increase of c-fos and Arc after a neuronal stimuli can occur already after 15 min and last for many hours (Katche et al., 2010; McReynolds et al., 2010), we studied both short (1 h) and long (24 h) term PA paradigms. No changes in expression of these genes were found in response to the short term paradigm. However, the long term paradigm decreased Arc expression in the PFC and increase c-fos expression in the CPu. Interestingly, both these changes were counteracted by treatment with WAY208466. The long term paradigm of PA by itself had no effects on Arc and c-fos in hippocampus, but WAY208466 caused a significant increase of both these genes in this region. Our data is in agreement with previous data showing increased hippocampal and cortical Arc expression in animals treated with another 5-HT6 agonist, LY586713 (de Foubert et al., 2007). The 5-HT6 receptor is, indeed, coupled to Gαs proteins, which stimulate adenylate cyclase and downstream signaling mechanisms (Yun et al., 2007; Riccioni et al., 2011). It is therefore possible that some of the IEG activation seen here is a direct action of 5-HT6 agonism on hippocampal and cortical neurons. However, since these brain regions express relatively low levels of 5-HT6 receptors, it is also likely that these Arc and c-fos responses reflect indirect activation. There are dense projections from the midbrain to the PFC and hippocampus (Ongür and Price, 2000) and there are multisynaptic loops interconnecting ventral striatum, where 5-HT6 receptors are very high, with the PFC and hippocampus. Because both 5-HT6 agonists and antagonists are procognitive in several memory tasks, it would be interesting to compare their effects on IEG expression. To our knowledge, there are no publications describing IEG expression after treatment with a 5-HT6 antagonist.

## Conclusion

WAY208466 facilitated emotional learning and induced antidepressant-like, but not anxiolytic, actions. Moreover, this 5-HT6 agonist stimulated molecular changes relevant for neuronal plasticity and memory formation in CPu, PFC and hippocampus. As noted above, the associations between behavioral responses and the molecular markers reported here are strictly correlational. To establish a causal relation between these events, experiments using gene knockouts would be necessary. Nonetheless, these data further emphasize an important role of 5-HT6 receptors in the regulation of neuronal signal transduction in relation to mood and cognition.

## Author Contributions

MP performed most of experiments, analysis, and writing. BM performed the immunoblottings, analyzed data and wrote the manuscript. RA contributed to the interpretation of the data and writing of the manuscript. PS designed the study and wrote the manuscript.

## Conflict of Interest Statement

The authors declare that the research was conducted in the absence of any commercial or financial relationships that could be construed as a potential conflict of interest.
